# Tuneable interplay between atomistic defects morphology and electrical properties of transparent p-type highly conductive off-stoichiometric Cu-Cr-O delafossite thin films

**DOI:** 10.1038/s41598-020-58312-z

**Published:** 2020-01-29

**Authors:** Petru Lunca-Popa, Jacques Botsoa, Mounib Bahri, Jonathan Crêpellière, Pierre Desgardin, Jean-Nicolas Audinot, Tom Wirtz, Didier Arl, Ovidiu Ersen, Marie-France Barthe, Damien Lenoble

**Affiliations:** 1 Material Research and Technology Department (MRT), Luxembourg Institute of Science and Technology (LIST), 41, rue de Brill, Belvaux, L-4422 Luxembourg; 2 Conditions Extrêmes et Matériaux: Haute température et Irradiation (CEMHTI) CNRS UPR 3079 - Site Cyclotron, 3A rue de la Férollerie, Orléans cedex 2, 45071 France; 3 Institut de Physique et Chimie des Matériaux de Strasbourg (IPCMS), UMR 7504 CNRS – Université de Strasbourg, 23 rue du Loess, Strasbourg Cedex 2, 67034 France

**Keywords:** Materials science, Nanoscience and technology, Physics

## Abstract

Off-stoichiometric copper chromium delafossites demonstrate the highest values of electric conductivity among the p-type transparent conducting oxides. Morphological and structural changes in Cu_0.66_Cr_1.33_O_2_ upon annealing processes are investigated. Chained copper vacancies were previously suggested as source of the high levels of doping in this material. High resolution Helium Ion Microscopy, Secondary Ion Mass Spectrometry and Transmission Electron Microscopy reveal a significant rearrangement of copper and chromium after the thermal treatments. Furthermore, Positron Annihilation Spectroscopy evidences the presence of vacancy defects within the delafossite layers which can be assigned to the Cu vacancy chains whose concentration decreases during the thermal process. These findings further confirm these chained vacancies as source of the p-type doping and suggest that the changes in electrical conductivities within the off-stoichiometric copper based delafossites are triggered by elemental rearrangements.

## Introduction

P-type transparent conductive oxides (TCOs) have lately attracted attention due to their promising applications in various fields such as flat panel displays, solar cells, photovoltaics and transparent electronic devices. The development of such material, with properties similar to its n-type counterpart (electric conductivity 10^3^ S cm^−1^, optical transmittance in the visible range well above 80%) will unlock the fabrication of a complete transparent active device (like a diode or a transistor)^1,2^. Among the others TCOs, copper based delafossites (with general formula CuM^3+^O_2_, where M = Al, Cr, B, Ga or In) have shown attractive performances with a record value of electric conductivity of 280 S cm^−1^ (with a visible transmittance of 50%)^3,4^. In such delafossites, the mixing of Cu^3d^ states and O^2p^ states forming the valence band leads to higher holes mobility. Tailoring the edge of the valence band by mixing molecular orbitals of the cations was proposed by Hosono and co-workers and demonstrated for CuAlO_2_ as the first p-type transparent delafossite^5,6^. Among delafossite materials, a special focus was given to CuCrO_2_ due to its highest density of 3d cations near the maximum of valence band and the covalent mixing between chromium and oxygen ions^7^. These two properties shall promote larger holes mobility and hence greater conductivities are expected for this material. However, intrinsic CuCrO_2_ presents a relatively low conductivity (10^−4^ S cm^−1^) and doping is required for obtaining decent electrical conductivity larger than 1 S cm^−1^ ^8^. Higher values started to be lately reported for delafossite films demonstrating peculiar off-stoichiometric ratios. Tens of S cm^−1^ and visible transparencies up to 60% were reported for off-stoichiometric copper chromium delafossite presenting up to 60% copper deficiency^9,10^. Furthermore, the synthesis of Cu-Cr-O delafossite thin films showing simultaneously a 33% copper deficiency and an excess of 33% chromium (i.e. Cu_0.66_Cr_1.33_O_2_), was reported^11,12^. The electrical conductivity of 102 S cm^−1^ measured in this case, constitutes a record for a intrinsically doped delafossite. It is only one order of magnitude lower than the conductivity of actual standard n-type semiconductors. Charge carriers’ densities beyond 10^21^ cm^−3^ were determined for this material. A new structural defect, consisting in chains of copper vacancies was observed whilst recent theoretical models have shown that in degenerated semiconductors the formation of such defects might be expected^13^. This defect was held responsible for the high level of p-type doping, as its healing after thermal treatments was associated with a five orders of magnitude decrease in electrical conductivity. Nevertheless, the delafossite phase, the average chemical environment and the peculiar stoichiometry remain unaltered upon thermal annealing and thus are not related directly to the electrical properties^14,15^. Short-range structural changes were considered as responsible for the significant changes in conductivity. This hypothesis is brought again into discussion in the present work. A new powerful instrument, a Helium Ion Microscope (HIM) coupled with a Secondary Ion Mass Spectrometer (SIMS) is used in order to elucidate more aspects related to the source of doping within the off-stoichiometric Cu-Cr-O delafossite thin films^16,17^. The HIM exhibits substantially higher resolution (0.5 nm) and surface sensitivity than a standard Scanning Electron Microscope (SEM) or Focused Ion Beam (FIB) system. The SIMS system coupled to the HIM, specifically developed at LIST, is capable of producing elemental maps with a lateral resolution of sub-20 nm approaching the physical resolution limit. In addition, the high-resolution Transmission Electron Microscopy (TEM) and Energy Dispersive X-ray (EDX) provide complementary information regarding the crystallographic structure, the presence of structural defects and the elemental map at the nanoscale. These investigations evidenced a significant elemental rearrangement occurring upon thermal treatments. Further analysis was performed using Positron Annihilation Spectroscopy, an established technique for the detection of vacancy-type defects in materials^18^. The results are consistent with the Cu chained vacancies as the source of p-type doping within the off-stoichiometric copper based delafossites. The changes in electrical conductivity are triggered by compositional rearrangement mechanisms based on the dissolution of the chains of vacancies into single Cu vacancies migrating to grain boundaries.

## Experimental Results

### X-Ray diffraction

**Figure 1:**
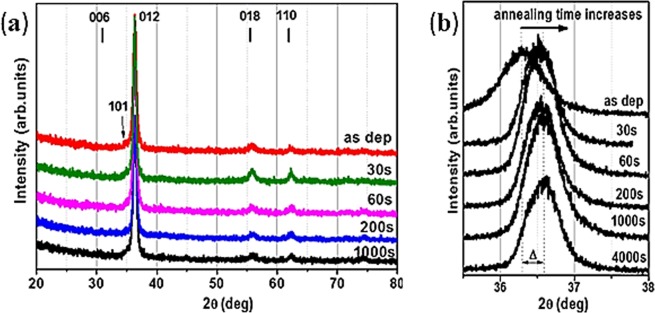
XRD diffractograms for as-deposited and annealed (at 900 °C) films and (deposited on sapphire). Times of annealing are indicated on the corresponding lines. (**a**) Grazing incidence diffractograms. The position of diffraction peaks for CuCrO_2_ (PDF 04–010–3330) is indicated; (**b**) High resolution scans around the 2θ position of the main (012) peak for as deposited and annealed films. The Δ symbol indicated the shift in 2θ position of the (012) peak.

Figure 1 depicts X-Ray diffractograms for as-deposited and for the Cu_0.66_Cr_1.33_O_2_ films annealed at 900 °C. The annealing time varied from 30 to 4000 s. For the phase identification (Fig. 1a), according to JCPDS-ICDD File card 04–010–3330, the main peak at 2θ = 36.4° can be attributed to the (012) crystallographic plane of copper chromium delafossite, with a small contribution of (101) plane at 2θ = 35.2°. Additional peaks attributed to the delafossite structure are positioned at 2θ values of 31.5° (006), 55.8° (018), 62.4° (110), 71.5° (116) and 75.5° (202). The delafossite structure is preserved after the thermal treatments, as no phase change is observed. High resolution X-Ray scans were then performed (Fig. 1b) around 2θ = 35.2°, the position of the (012) peak. A shift to the larger angles (corresponding to the detection of smaller inter-planar distances) is observed even after short thermal treatments^14^.

### Electron microscopy

**Figure 2:**
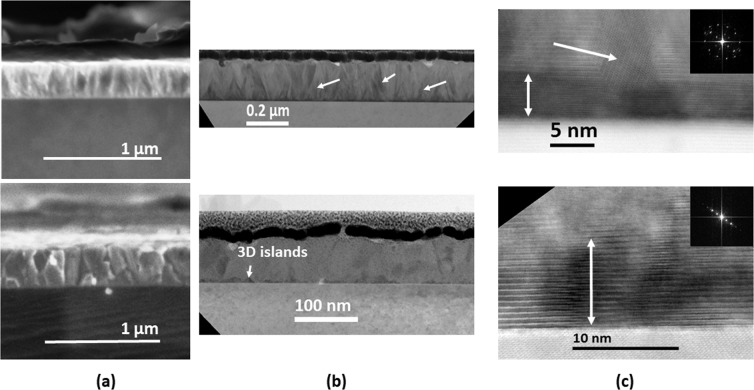
Electron microscopy analysis for as-deposited (top) and annealed (bottom) films. Annealing temperature: 900 °C. Annealing time: 1000 sec. (**a**) SEM micrographs; (**b**) Typical cross section scanning bright field TEM (BF-STEM). The arrows illustrate the presence of the structural defects in as-deposited films and the contrasted 3D islands in annealed film; (**c**) high resolution images. The arrows indicate the thicknesses of the epitaxial layer and a typical twinned domain is as-deposited film. Inset: corresponding FFT patterns.

Scanning electron microscopy (SEM) images of films deposited on Si reveal significant changes of the internal structure between as-deposited and annealed (1000 seconds at 900 °C) films (Fig. 2a). A columnar compact growth is observed in the case of as-deposited sample. The columns have a width of up to 50 nm while the length is covering the whole films’ thickness. The thermal treatment leads to an increase of the columns ‘diameter up to 200 nm. The analysis of the transmission electron microscopy images and corresponding elemental maps provide a more detailed insight on the internal structure of the layers. Figure 2b,c show global and high resolution STEM bright field images of the as-deposited (top) and annealed (bottom) samples. A high density of structural defects layer is observed across the Cu-Cr-O as-deposited sample. These defects have a spatial density of about 18 defects/µm and separate two twinned domains which can be assigned to vertical columns. In the annealed sample, the density of these defects is three times smaller and the width of the crystallographic domains (without defects) increases. All the domains are characterized by a high crystalline order and have the same crystallographic structure, before and after annealing. This is supported by the analysis of the high-resolution images and corresponding to Fourier transform patterns (the typical distance of the (012) plane of CuCrO_2_ structure is clearly visible in all the images). Regarding the interface with the substrate, a layer with a thickness of about 5 nm and made of several successive crystallographic planes is visible close to the substrate with a contrast slightly different from the remaining part of the sample. This layer corresponds probably to the adaptation of the CuCrO_2_ crystallographic arrangement to the epitaxial one on the substrate. Beyond this thickness the structural strains relax and lead to the bulk-like structure. After annealing some 3D islands with a typical height of 15 nm, characterized by a darker contrast, appear at the interface. This might be related to the rearrangement of the epitaxial layer by an atomic diffusion of some atoms along the crystallographic defects.

It is well known that such rearrangement processes at the interface with the substrate or more generally within the whole layers may lead sometimes to segregation effects. To solve this issue, we have analysed both specimens by EDX elemental mapping (Fig. S1) and observed no notable modification of the Cu and Cr distributions after annealing. Indeed, the elemental distributions of the Cr and Cu atoms seem to be homogeneous in both samples, though the thermal treatments lead to a clear rearrangement of the internal structure of the layer.

### Helium ion microscopy and secondary ion mass spectrometry

**Figure 3:**
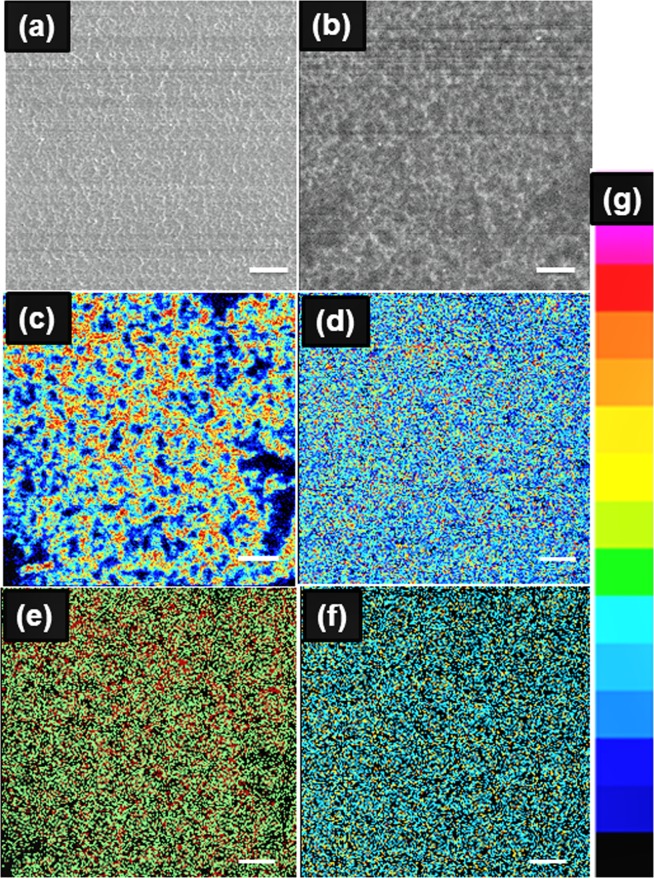
Comparison of Cu-Cr-O films before (**a**,**c**,**e**) and after (**b**,**d**,**f**) annealing at 900 °C for 2000 s. (**a**,**b**) Secondary electron (SE) images from Helium Ion Microscopy (HIM); Secondary Ion Mass Spectrometry (SIMS) before and after annealing for: (**c**,**d**) Chromium; (**e**,**f**) Copper. The scale bar corresponds to 500 nm for all micrographs; (**g**) the signal intensity scale for all micrographs. Low to high intensity - blue to red.

The surface morphology of the Cu_0.66_Cr_1.33_O_2_ films was observed by HIM. A surface pre-cleaning has been performed for 5 minutes using a 10 pA sputtering current. The as-deposited film has a homogeneous surface (as depicted in Fig. 3a), contrasting with a significant roughness observed after annealing (Fig. 3b). This top view is in a good agreement with the previous SEM cross section (lateral view) illustrated in Fig. 2a. The copper and chromium HIM-SIMS images for as-deposited and annealed films are presented in Fig. 3c–f. A mixture of areas with high (red) and low (dark blue) elemental concentrations are observed in the as-deposited films, as especially observed in the case of chromium (Fig. 3c). The thermal treatment has a levelling effect on concentration, (Fig. 3d,f) as the annealing sample depicts a uniform distribution, characterized by an overall moderated concentration (green–blue dots). The overlapped Cr/Cu SIMS image (Fig. S2) indicates a higher chemical blending (presence of the two elements at very similar location) within the annealed sample.

### Electrical properties

**Figure 4:**
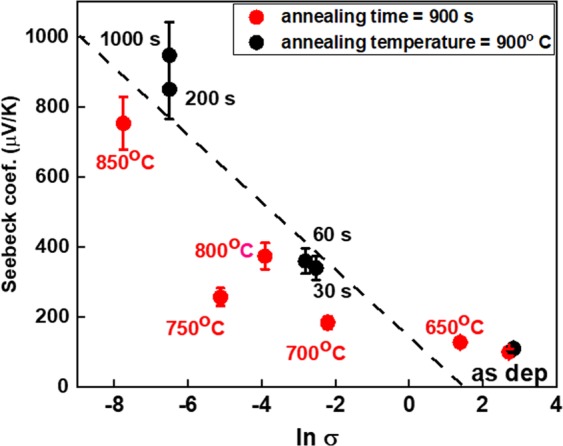
Jonker plot for Cu-Cr-O films annealed for various interval times at 900 °C (black circles) or at different temperatures for 900 s (red circles). Experimental data points from ref. ^15^. Numerical values in Table S1. The dashed Jonker line was drawn as eye guide.

A detailed analysis of electrical changes upon annealing processes has been presented elsewhere^15^. Changes in electrical properties are observed for Cu-Cr-O films heated at different temperatures for various time intervals. The electric conductivity decreases from values around 100 S cm^−1^ (as-deposited films) down to 10^−4^ S cm^−1^ after long annealing at high temperatures. Concurrently, the Seebeck coefficient increases from values around 100 µV K^−1^ for as deposited sample up to a maximal measurable value of 870 µV K^−1^ for the annealed samples. All experimental values are depicted in Fig. 4 by the mean of the Jonker plot, commonly used when electronic transport properties are studied^19^. The dashed line (Jonker line) has a slope of kB/q = ±86.17 µV K^−1^ and corresponds to a thermally activated non-degenerate semiconductor behaviour. As observed, a full range linear fit is not reliable in this case and the non-degenerate regime might be thus excluded. Indeed, the most frequent model used to describe the charge carriers’ transport mechanism in copper delafossites is the small polaron although the large polarons in highly-degenerate semiconductor shall not be fully excluded. In this case, the small polaron model was considered to calculate the charge concentration from the values of Seebeck coefficients (Table S1)^15^. The concentration of the holes decreases from 10^21^ cm^−3^ in as-deposited samples down to 10^16^ cm^−3^ in the most insulating annealed sample. This change might be further associated to the five orders decrease of measured electrical conductivity

### Positron annihilation spectroscopy

**Figure 5:**
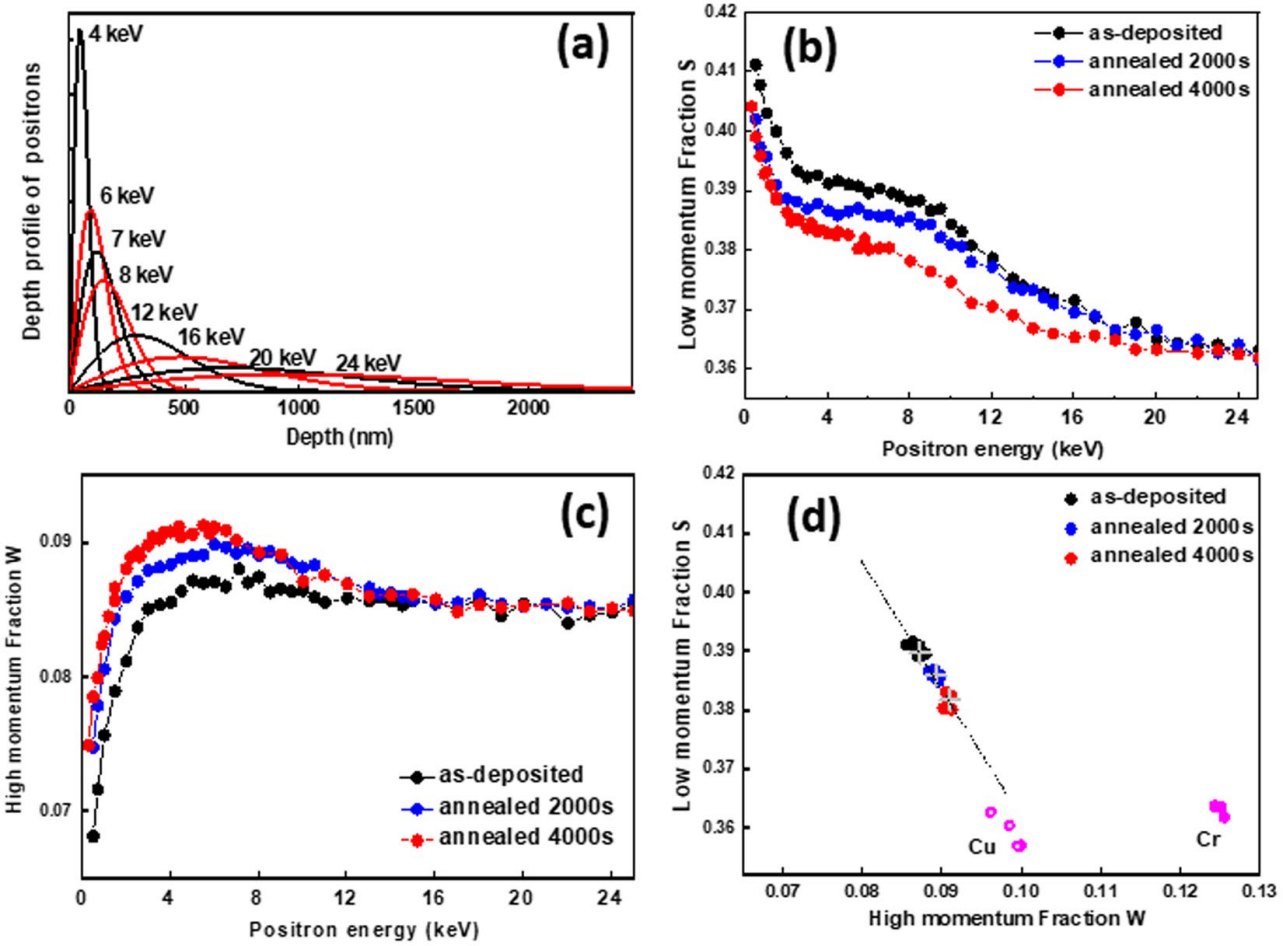
(**a**) Simulated positron implantation profile in CuCrO_2_ as a function of their incident energy; (**b**) Low momentum fraction S and (**c**) high momentum fraction W as a function of positron incident energy for an as-deposited sample and samples annealed for 2000 s and 4000 s respectively; (**d**) Corresponding S-W plot with added (S, W) points measured in the bulk of Cu and Cr.

Figure 5a shows the positron implantation profile in CuCrO_2_, which can be simulated by the Makhovian profile for different incident positron energies ranging from 4 to 25 keV^20^. If we consider a 380 nm-thick delafossite film, positrons are implanted almost exclusively in the film for incident energies below 7 keV. As the positron incident energy increases beyond this value, an increasing fraction of positrons will stop in the underlying sapphire substrate. The variations of S and W annihilation parameters (thoroughly described within the experimental methods section) with positron incident energy E for one as-deposited and two annealed samples (annealing times: 2000 s and 4000 s) are shown in Fig. 5b,c, respectively.

Within the 0–7 keV energy range, the measured S and W can be viewed as a combination of surface and bulk characteristics of the delafossite film. At the lowest energies, the contribution from the surface prevails (high S and low W values) and as the energy increases, this contribution decreases in favour of bulk annihilation characteristics. S-E and W-E profiles both demonstrate plateaus as from 4 keV. These plateaus indicate that the delafossite layers are homogenous, from a defect perspective. From these plateaus, we can observe that S parameter decreases while the W parameter increases upon annealing. Beyond 7 keV, contributions from annihilations taking place in the underlying substrate have to be taken into account and as the positron energy is increased, S and W values shift towards those of Al_2_O_3_ bulk (S = 0.360, W = 0.085).

The S-W plot, shown in Fig. 5d, is typically used to gain insight into annihilation states present in the sample. The (S, W) points measured in the bulk of Cu and Cr are also plotted on the same graph. Since we are mainly interested in the annihilations coming from the bulk of the delafossite layer, the corresponding (S, W) points shown here are those measured for positron incident energies between 4 and 7 keV. To check the suitability of these points as representatives of bulk annihilation characteristics in the films we have performed simulations of the S-E and W-E data using the Variable Energy Positron Fit (VEPFIT) software by considering a multi-layer model^20^. The best fit in each case is given by a two-layer model where the first layer is the delafossite film, and the second layer being Al_2_O_3_bulk. The (S, W) points inferred from VEPFIT for the first layer are presented as a cross for each sample and they are seen at the centre of the scattered points measured in the energy range from 4 to 7 keV. This confirms that these points feature annihilation mechanisms within the bulk of the delafossite layer. One can notice that these points are all aligned on a straight line (see the dotted line drawn). It is also interesting to notice that the effective positron diffusion lengths deduced from VEPFIT in the delafossite layers is around 10–20 nm (Table S2) which is one order of magnitude lower than typical values measured in defect-free semiconductors (200–300 nm).

## Discussions

Results from this paper are strongly correlated with our previous findings^12,14,15^. Henceforth, as-deposited films present electrical conductivities around 100 of S cm^−1^, charge carriers’ concentration of 10^21^ cm^−3^ whilst the Fermi level is situated extremely close to the valence band. Within these films, chained copper vacancies were observed at the same time with the chromium excess distributed within the bulk of crystalline grains. Thermal treatments lead to a severe drop of electrical conductivity down to 10^−4^ S cm^−1^ associated with a decrease of holes’ concentration down to 10^16^ cm^−3^. The copper chained vacancies are healed during such treatments. The SEM micrographs reveal an increase of the grains’ size (Fig. 2a) upon annealing while a small shift of the diffraction peaks towards larger 2θ is observed (Fig. 1c). This indicates a relaxation of the c lattice parameter (direction of O-Cu-O bonds), which can be further correlated with the crystallographic reorientation evidenced by TEM analysis (Fig. 2b,c). The STEM analysis demonstrates that the density of structural defects decreases and the width of coherent crystallographic domains increases. This rearrangement of the whole layer through local atomic diffusion which is enhanced by the important number of copper vacancies in as-deposited sample. On the other hand, XPS, Raman and XRD analyses do not reveal other significant changes on chemical environment. The delafossite phase is preserved while the peculiar 0.66:1.33:2 stoichiometry remains unaltered. The volume probed with these investigation techniques depends on the beam cross-section, usually few squared microns. It is obvious then, that changes occurring at the grain size level (a few tens of nanometres for as-deposited films), cannot be detected likewise and higher resolution techniques must be used. This advocates that changes might occur at nanoscale, beyond the resolution of methods mentioned above. We therefore engaged Helium Ion Microscopy with the results depicted in Fig. 3. Taking benefit of its superior spatial resolution and surface sensitivity we were able to evidence a clear elemental rearrangement occurring upon the thermal treatment. Within the as-deposited films, the existence of the chained copper vacancies and the distribution of chromium in excess inside the grains induce a non-homogenous overall repartition of atoms within the material. This is evidenced within Fig. 3c–e. In the as-deposited sample, the chromium and the copper elemental distributions are non-homogenous, showing a mixture of areas with low concentrations (dark blue) and high concentrations (red). After the thermal treatment this distribution becomes uniform, characterized by moderate atomic concentration (green dots). We previously proposed that the changes in electrical conductivities in this system are triggered by Ostwald ripening mechanisms based on the dissolution of the chains of copper vacancies^14^.

In order to elucidate this aspect, PAS measurements were performed on an as-deposited film and on films annealed for 2000 s and 4000 s respectively. The main results are presented in Fig. 5. Upon annealing, there is a decrease in the S parameter measured in the delafossite layer along with an increase in the W parameter. From the S-W plot, one can observe that the (S, W) points for the three films are collinear. This behaviour can be explained in the scheme of two distinct annihilation states contributing to the overall annihilation characteristics measured. One of the states corresponds to the positron annihilation in the untrapped state in the CuCrO_2_ lattice with corresponding annihilation characteristics (S_L_, W_L_), and the other state corresponds to positrons annihilating while being trapped in a single type of vacancy defect, referred to as V_i_ (where *i* is the number of vacancies in the defect), with corresponding annihilation characteristics (S_Vi_, W_Vi_). Within this model, (S_L_, W_L_) and (S_Vi_, W_Vi_) are aligned on the dotted line shown in S-W plot. However, at this stage it is not possible to position these points along the dotted line. Nevertheless, the (S, W) values measured in the bulk of a Cu and Cr sample and presented on the same plot allow us to deduce that (S_L_, W_L_) range in the lower right side of the (S, W) points measured in the delafossite films. Within this picture, the shift in (S, W) upon annealing can be viewed as a decrease in concentration of V_i_. Based on previous observations, we propose that V_i_ refers to the chained copper vacancy. However, it must be argued that a decrease of V_i_ upon annealing by the shortening of the vacancy chains induced by Ostwald ripening mechanisms will imply the existence of different “sizes” of Cu vacancy chains in the annealed samples and hence the one trap model can no longer be applied. However, it has been shown from theoretical studies performed in Si that the (S, W) points for different vacancy clusters are almost collinear with S increasing (and W decreasing) with the size of vacancy clusters^21^. In our case, this will entail that the dissolution of the vacancy chain would still be observed as a linear shift of (S, W) towards (S_L_,W_L_). It must be added that the dissolution is not complete even for the film annealed for 4000 seconds as it still shows a very weak electrical conductivity (10^−4^ S cm^−1^), which might suggest the presence of a small amount of vacancies, having possibly small cluster morphology. Finally, the shift tendency of the (S, W) points for the annealed films is towards those measured in pure copper. This suggests that the chemical environment at positrons annihilation sites becomes enriched in Cu after annealing as could be expected in the case of a decrease in size of the Cu vacancy chains.

## Conclusion

In conclusion, this work fully demonstrates that chained copper vacancies as the source of p-type doping in Cu_0.66_Cr_1.33_O_2_ thin films deposited by chemical vapour deposition. Upon thermal annealing an important atomistic rearrangement of atoms and defects occurs leading to the shrinkage of chains of vacancies until almost their extinction. This suggests the presence of a ripening mechanisms based on the dissolution of the chains of copper vacancies being healed on grain boundaries during the demonstrated grain growth and leading to the drop of electrical conductivity in annealed films. Investigations related to charge transport mechanisms, and in particular on the effective mass of holes and their scattering mechanisms, are needed for a further tuning of electric properties.

## Methods

### Films deposition

Thin films were deposited on Al_2_O_3_ c-cut and on Si (100) substrates using a Dynamic Liquid Injection - Metal Organic Chemical Vapor Deposition system (DLI-MOCVD, MC200 from Annealsys). Copper bis (2, 2, 6, 6-tetramethyl-3, 5-heptanedionate) and Chromium (III) tris (2, 2, 6, 6-tetramethyl-3, 5-heptanedionate) were used as precursors using a 2.5 mM equimolar Cu/Cr solution in cyclohexane. The deposition parameters are: temperature substrate = 450 °C; oxygen flow = 2000 standard cubic centimetres per minute (sccm); nitrogen flow = 850 sccm; total process pressure = 12 mbar. A detailed description of the deposition approach fabrication can be found elsewhere^12^. The annealing processes were performed in a Rapid Thermal Annealing reactor (Annealsys) in same gaseous conditions as during the deposition.

### X-Ray diffraction (XRD)

The crystal structure was investigated using a Brucker D8 Discover diffractometer with Cu K alpha radiation of 0.154 nm operating at 40 kV and 40 mA in parallel beam configuration. Grazing incidence at an angle of 0.5° was used as investigation method.

### Scanning electron microscopy (SEM)

The cross section of Cu_0.66_Cr_1.33_O_2_ films deposited on Si was inspected by Scanning Electron Microscopy using a FEI Helios 50 High Resolution system. Through-Lens-Detector (TLD) in Secondary Electron Mode imaging was engaged.

### Transmission electron microscopy (TEM) and energy dispersive X-ray (EDX) spectroscopy

The TEM analyses in the scanning mode (STEM) combined with the elemental mapping by EDX were performed on a Cs corrected JEOL 2100 F microscope operating at 200 kV. The elemental maps have been acquired in the scanning mode by using an SDD detector (DrySD60GV from JEOL) having with a solid angle of 0.5 srad. The analysed CuCrO_2_ specimens were prepared by Focused Ion Beam (FIB) technique. The density of the structural defects along the Cu-Cr-O layer were evaluated by using an image software (Digital Micrograph from Gatan). The defects were accounted from the middle of the analyzed areas in several representative images; by dividing the as-obtained number by the whole corresponding distance, an average density of defects was obtained.

### Helium ion microscopy (HIM)

*Secondary* electron images were obtained with an ORION NanoFab Helium Ion Microscope (HIM) (ZEISS, Peabody, USA). Helium Ion Microscopy is based on the detection (by a conventional Everhart-Thornley detector) of the secondary electrons emitted from the sample surface scanned by a focused energetic He^+^ or Ne^+^ ion beam. In this study, the images were acquired with a 20 keV He^+^ beam of 1 pA for a matrix of 1024 × 1024 pixels and a counting time of 5 µs per pixel. Furthermore, analytical information was obtained thanks to the Secondary Ion Mass Spectrometry (SIMS) system specifically developed for the HIM^16,17^. This system has been optimized for the collection of the secondary ions generated by the primary ion bombardment (Ne^+^, 20 keV, 3 pA), their transmission to the high-performance double focusing magnetic sector mass spectrometer and their detection in a multi-collection system (4 Channel Electron Multipliers). For the analysis of the Cr-Cu samples the instrument was tuned for the detection of the chromium (^52^Cr, m = 51.941 amu) and copper (^63^Cu, m = 62.930 amu) isotopes in a matrix of 512 × 512 pixels with a counting time of 5 ms per pixel.

### Positron annihilation spectroscopy (PAS)

The samples were characterized by using a slow positron beam coupled to a Doppler broadening spectrometer (SPB-DB) available at the CEMHTI laboratory. A comprehensive description of the experimental setup and the basics of this powerful method are well described in ref. ^22^. A monoenergetic positron beam, with a diameter of 3 mm, was generated from a ^22^Na source. The positron beam energy was varied in the range of 0.5 to 25 keV. The spectrum of the γ-ray annihilation photons (centred at 511 keV) coming from the sample is recorded using a high resolution gamma spectrometer equipped with a Germanium detector (1.24 keV resolution at 514 keV). This Doppler broadened spectrum is characterized by two line shape parameters: ***S*** and ***W***. ***S*** is defined as the ratio of counts in the central region of the spectrum to the total counts, represents the fraction of positron-electron pairs annihilated with low momentum and is thus related mostly to annihilations with valence electrons. ***W*** is defined as the ratio of counts in the wing regions of the spectrum to the total counts, represents the fraction of positron-electron pairs annihilated with high momentum and is thus more specifically related to annihilations with core electrons. For our experiments, the momentum ranges for the calculation of ***S*** and ***W*** are 0 − |2.80| × 10^−3^ m_0_C and |10.61| − |26.35| × 10^−3^ m_0_C respectively. The sensitivity of the PAS technique to the type and concentration of vacancy defects in solids reside in the fact that positrons can be especially trapped in these defects, where the electron density is low, before annihilating. When positrons are trapped at vacancies, their smaller overlap with core electrons narrows the positron-electron momentum distribution resulting in an increase of S and a reduction of W. Hence S and W yield information about the presence of vacancy defects in solids. For this study, PAS measurements were performed on the bare Al_2_O_3_ substrate, on as-deposited and thermally treated samples.

### Electrical properties

The electric resistivity was measured using four probes in linear configuration. The distance between probes was 1 mm. The Seebeck coefficient was measured in a homemade system using a copper wire as reference. The samples deposited on sapphire were cut in 7 mm × 7 mm pieces and the four probes were positioned as far as possible from the edges. The finite samples’ size effect was accounted by applying geometric correction factors.

## Supplementary information

Supplementary information.
