# Supplementary material for: Tuneable interplay between atomistic defects morphology and electrical properties of transparent p-type highly conductive off-stoichiometric Cu-Cr-O delafossite thin films
Source: Sci Rep. 2020 Jan 29;10:1416. doi: 10.1038/s41598-020-58312-z (PMC6989665; doi:10.1038/s41598-020-58312-z)
Supplement: Supplementary file 1 — Supplementary information. [file 41598_2020_58312_MOESM1_ESM.pdf]

Supplementary Information

Tuneable interplay between atomistic defects morphology and electrical properties of transparent p-type highly conductive off-stoichiometric Cu-Cr-O delafossite thin films

Petru Lunca-Popa*, Jacques Botsoa, Mounib Bahri, Jonathan Crêpellière, Pierre Desgardin, Jean-Nicolas Audinot, Tom Wirtz, Didier Arl, Ovidiu Ersen, Marie-France Barthe, Damien Lenoble

STEM-EDX Elemental mapping for as-deposited and annealed samples

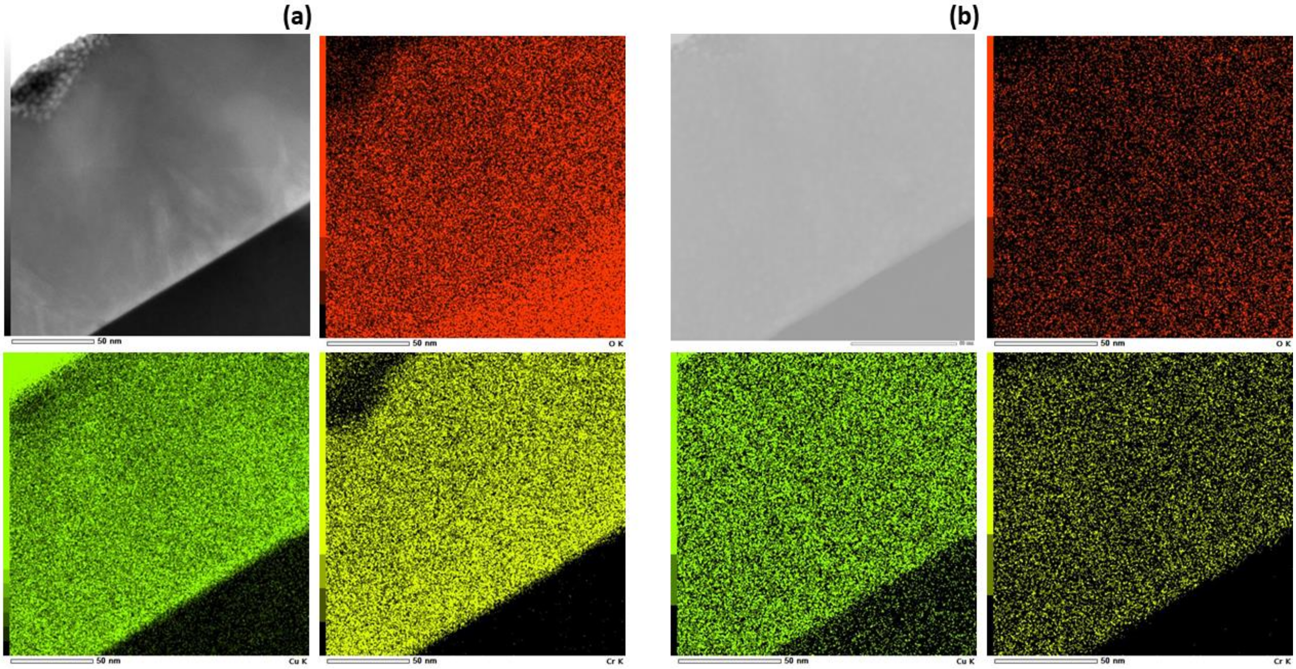

***Figure S1**: Elemental maps obtained by STEM-EDX on the as deposited sample (a) and annealed one (b). The elemental maps for O are in red, for Cu in green and for Cr in yellow, respectively. Scale bars: 50 nm. Copper TEM grid were used*

Seebeck coefficient expression within small polaron model

$$S = \frac{k_B}{q} ln\left[\left(\frac{g_1}{g_2}\right)\frac{[Cu^+]}{[Cu^{2+}]}\right] \quad \text{(Eq. S1)}$$

where k_B is the Boltzmann constant, h is the Planck constant, q is the carriers' charge, g_1 and g_2 are the electron degeneracy of Cu^+ and Cu^{2+}, respectively, and [Cu+] and $[Cu^{2+}]$ are ionic proportions.

Table S1. Measured electrical conductivities, measured Seebeck coefficients and calculated carriers' concentration for Cu-Cr-O films annealed for various time intervals at different temperatures

Electrical conductivity (S cm^{-1})	Seebeck coefficient ($\mu V \cdot K^{-1}$)	Carrier concentration cm^{-3}	Temperature of annealing (°C)	Annealing time (s)
15	100	$1.7\ 10^{21}$	-	0
4	128	$1.2\ 10^{21}$	650	3600
0.11	184	$6.6\ 10^{20}$	700	900
0.006	257	$2.9\ 10^{20}$	750	900
0.002	374	$7.3\ 10^{19}$	800	900
0.0004	753	$9.3\ 10^{17}$	850	900
17	110	$1.5\ 10^{21}$	-	0
0.08	340	$1.1\ 10^{20}$	900	30
0.06	360	$8.9\ 10^{20}$	900	60
0.0015	850	$3.0\ 10^{16}$	900	200
0.0012	947	$9.4\ 10^{16}$	900	1000

The SIMS overlaid Chromium (green)/ Copper (red) image

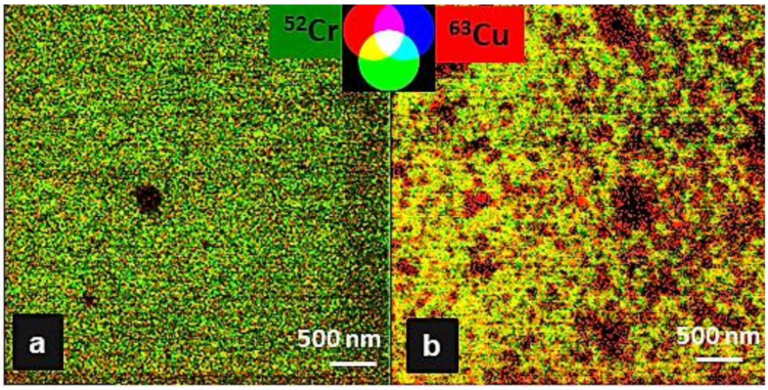

Figure S2:

Comparison of Cu-Cr-O films before and after annealing at 900 °C for 2000s.

(a, b) Overlay of the Secondary Ion Mass Spectrometry (SIMS) imaging of chromium (green) and copper (red) before and after annealing. The overlay of red and green provides a yellow color (inset of d). The scale bar corresponds to 500 nm.

The overlay of the Chromium image (green) and Copper image (red) obtained by SIMS performed on the HIM allows the evaluation of the chemical blend. Before annealing, the both elements do not seem to be co-localized (figure a). In the annealed film, the yellow color (corresponding to the sum red and green) predominates (figure b), highlighting the presence of the two elements at very similar location (within the same pixel). Some overlapping between

areas containing Cu and areas with high concentrations of atoms is expected for this image as both are associated with red color.

Table S2. S, W and effective positron diffusion length ($\boldsymbol{L_+^1}$) values obtained from VEPFIT for the delafossite layer in the as-deposited sample and samples annealed for 2000 and 4000s

**Film**	**S**	**W**	$\boldsymbol{L_+^1}$ $(\boldsymbol{nm})$
As-deposited	0.3897	0.0873	18.8
2000 s annealed	0.3859	0.0893	15.4
4000s annealed	0.3817	0.0910	10.2
